# Chest auscultation – tradition and expectations *Concerns and promises in the era of smart stethoscopes*

**DOI:** 10.1080/02813432.2026.2739502

**Published:** 2026-09-26

**Authors:** Hasse Melbye, Carl Edvard Rudebeck, Hans Pasterkamp

**Affiliations:** ^a^ General Practice Research Unit, Department of Community Medicine, Faculty of Health Sciences, UIT the Arctic University of Norway, Tromsø, Norway; ^b^ UiT Norges arktiske universitet, Västervik, Sweden; ^c^ Department of Pediatrics and Child Health, Max Rady College of Medicine, University of Manitoba, Winnipeg, Canada

**Keywords:** Auscultation, stethoscope, heart sounds, lung sounds, chest diseases

## Abstract

AI-assisted digital stethoscopes can classify heart and lung sounds with accuracy comparable to or exceeding that of the average clinician and help the physician in the diagnostic process. With this essay we want to discuss benefits and downsides of this new technology. Will proficiency in auscultation, which brings joy and pride to many stethoscope users, be suffering when interpretation of chest sounds is taken over by AI? And how will recommendations from AI in the diagnostic process influence decision making and the patient-doctor relationship? A tendency toward deskilling of auscultation proficiency can be counteracted when AI-assisted interpretation is used as a reflective tool, comparing machine output against clinical perception to sharpen diagnostic acuity. This concept of a collaboration of humans and AI will be important with regard to keeping AI-algorithms trustworthy. We still need more research on heart and lung sounds, to gain a detailed understanding of the clinical significance of various sound characteristics. The smart stethoscope will be useful for this research. Better knowledge will benefit not only algorithm development but also clinicians using traditional stethoscopes. The traditional stethoscope will probably prevail as the main instruments for chest auscultation in foreseeable future. Smart stethoscopes may become useful tools in the education of their users.

## Introduction

A 35-year-old woman attended a GP’s office due to a cough and shortness of breath that had lasted for several days. She had heard wheezing from her chest, and, with her mobile phone, she had recorded these sounds. The GP, who had never before experienced a patient presenting recordings of their body sounds, recognized the sounds as typical of asthma. With a traditional stethoscope, he could appreciate her breathing problems; expiratory wheezes were clearly audible. No surprise, but for the GP a welcome confirmation.

This event illustrates how chest auscultation is typically used by GPs today to obtain a more direct impression of the patient’s cardiorespiratory system. The event also illustrates changes we can now anticipate for chest auscultation. New electronic stethoscopes can record chest sounds [1], and integrated algorithms can classify heart and lung sounds more reliably than the average clinician [2–5]. Interpretation of the sounds done by artificial intelligence (AI) may help the physician in the diagnostic process [1]. Such AI-assisted digital stethoscopes, also called ‘smart stethoscopes’, may be useful in medical education. Improved teaching of clinical skills have been widely called for [6]. In the clinic, chest sounds can be recorded and replayed and sound features be demonstrated. Learners will receive prompt feedback on their own interpretations.

The 35-year-old woman with possible asthma also shows how patients are taking a more active role to assist in the diagnosis of their illness. Patients show the doctor pictures of their rashes and also what the internet says about their symptoms. Smart stethoscopes will become one of several tools supporting lay people in managing their own health.

With this essay we want to discuss benefits and downsides of this new technology. The authors are one professor emeritus of general practice (HM) and one professor emeritus of pediatrics (HP), both experienced in research on chest sounds, and one previous professor of general practice (CER) with a research history focusing on the doctor-patient relationship. We ask whether proficiency in auscultation, which brings joy and pride to many stethoscope users, will be suffering when interpretation of chest sounds is taken over by AI, and how recommendations from AI in the diagnostic process will influence decision making and the patient-doctor relationship.

## Material and methods

We present elements of the history of chest auscultation and research on its diagnostic usefulness. Regarding the impact of the smart stethoscope, we also refer to opinion leaders in the field. This paper is based on our own research and literature search on PubMed and with AI assistance from Claude Sonnet 4.6 (Anthropic, accessed *via* claude.ai)

## Rise and fall of the stethoscope

The traditional stethoscope, more than 200 years old, remains in use by physicians, nurses, and paramedics worldwide. In its early years, the rapid spread and success of the instrument were facilitated by research conducted by its inventor. The French physician RTH Laennec correlated the sounds heard from his patients’ chests with specific pathological findings identified at autopsy [7]. When applied to patients with a possible heart or lung disease, a more certain diagnosis could be made, and treatment given on a stronger empirical basis. A new routine in the doctor-patient encounter was established: Whenever the patient complained of symptoms from the chest, such as coughing, shortness of breath, or palpitations, chest auscultation became mandatory. The possibility of revealing diseases like valvular heart disease and obstructive lung disease by listening to the chest in apparently healthy individuals also paved the way for auscultation as part of routine health examinations.

After the invention of the stethoscope, there was a long period when chest auscultation was the main diagnostic method for chest diseases. Severe chest diseases were commonplace in both young and old, and pneumonia and pulmonary tuberculosis detected by hearing abnormal (adventitious) lung sounds were leading causes of death. Rheumatic fever after streptococcal infection induced heart valve disease, which could be identified by characteristic heart murmurs [8]. The burden of these diseases has been declining in the western world, and chest imaging, based on radiology and ultrasound technology, has replaced auscultation in hospitals and secondary care clinics. We have seen a decline in auscultation skills among medical students and hospital doctors [9], and clinicians have recommended replacing the traditional stethoscope by modern imaging [10]. However, while the standing of the stethoscope was dropping, developments in the field of acoustics led to a new interest in chest sounds. Acoustic analysis of recorded lung sounds gave new insight into how abnormal sounds are generated [11], and electronic and digital versions of the old instrument with enhanced acoustic properties came on the market. In the last decade, there has been a sharp rise in the number of scientific publications on chest sounds due to the implementation of machine learning (Figure 1). Computers have been trained to identify and interpret abnormal chest sounds. AI-assisted stethoscopes are now a reality. Clinicians can be told which sounds they are hearing, and what the possible diagnostic implications are. The stethoscope may thus be entering a new era of clinical relevance.

**Figure 1.**
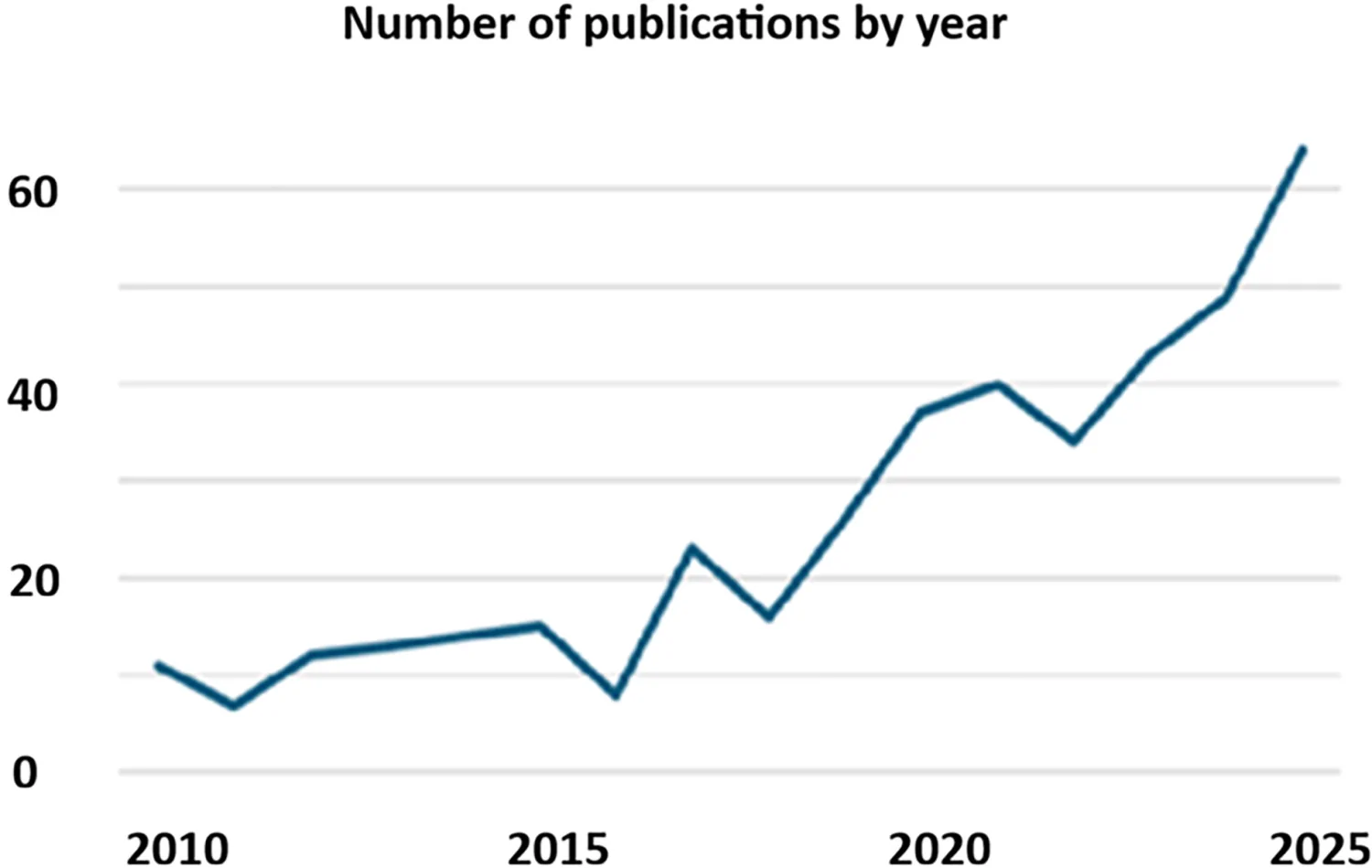
Number of publications on chest auscultation registered in PubMed between 2010 and 2025. Search line: “chest auscultation” OR “digital stethoscope” OR “smart stethoscope” OR (AI AND auscultation)

## Occurrence of abnormal chest sounds

Before further discussing the impact of the smart stethoscope, it is necessary to consider what we know about the diagnostic usefulness of the traditional stethoscope. The sounds generated in the lungs are influenced by the depth and speed of breathing. The normal breath sound is heard over a healthy lung, and when it is diminished, emphysema, pneumonia, or air or fluid in the intrapleural space can be suspected [11]. Abnormal lung sounds appear when there is a disturbance of the airflow in the bronchial tree. Wheezes and rhonchi are generated when air passes through narrowed bronchi, whereas crackles appear when closed airways suddenly open [11]. Wheezes and rhonchi are typically heard in acute bronchitis, asthma and chronic obstructive pulmonary disease (COPD), and not infrequently in asymptomatic smokers [11]. In children, wheezes may indicate asthma, while the snore-like sound of rhonchi, which arises from the movement of air through secretions, is particularly common in young children with increased bronchial secretions (‘snotty lungs’) [12]. Wheezes were heard in 18% of participants in Tromsø 7, a large population-based health survey of adults between the ages of 40–99 years, whereas crackles were heard in 13% [13]. Crackles occurred four times more frequently in participants aged 70 years or more than in those aged 40–49 years, 20% and 5%, respectively [13]. Lung crackles can also be associated with heart diseases, when cardiac failure causes pulmonary congestion [14]. In chronic heart failure crackles are often absent, and biomarkers mirroring heart strain, such as natriuretic peptides, are more useful for the diagnosis [15]. Auscultation of the heart provides complementary information on heart rhythm and the presence of murmurs. Heart murmurs are mainly caused by turbulent blood flow through a heart valve, which is sometimes associated with severe heart valve disease (VHD) and heart failure [16].

When interpreting an abnormal auscultatory finding, it is important to consider how often it occurs in subjects with and without the diseases in question. To illustrate this point, we can look at the association between crackles and pneumonia. Crackles have been regarded as a typical sign of pneumonia and a reason to prescribe antibiotics in patients presenting with cough [17,18]. In a study by van Vugt et al. of 2820 patients in primary care with acute cough, crackles were heard in 59% among the 140 participants with X-ray detected pneumonia and in 7% among the 2670 without [19]. A lower frequency (sensitivity) of crackles for pneumonia has been reported in other studies; Diehr et al. found a sensitivity as low as 19% [20]. In a study of hospitalized patients with radiographically proven pneumonia, repeated auscultation revealed crackles in 50%, but in half of these the crackles were heard over lung sections where no radiographic infiltrate could be seen [21]. No wonder that primary care doctors have called for tests that can identify pneumonia with higher sensitivity and specificity, such as inflammation markers [19].

The great variation in detection of crackles in patients with pneumonia can partly be explained by variations in the disease. The depth and rate of the patient’s breathing during auscultation also influence the generation of sounds and the listener’s ability to discern crackles from normal breath sounds [22]. A third reason for the variation is the difference in how the listeners interpret what they hear. The interobserver variation is often considerable when health workers rate the same lung sound recordings [23]. The incomplete standardization of terminology for lung sounds across languages adds to the misunderstanding [24]. This variation weakens the association between crackles and pneumonia measured in clinical studies. The association is further affected by the background prevalence of crackles in patients without pneumonia. As patients get older there is an increasing probability that crackles are attributable to other conditions [13]. In the clinic, it is often a challenge to differentiate between pneumonia and an acute exacerbation of chronic obstructive pulmonary disease (COPD), particularly since crackles become more prevalent during COPD exacerbations [25]. However, crackles are not homogeneous, and trained auscultators are often able to differentiate between the typical coarse and early inspiratory crackles in COPD and the fine and late inspiratory crackles heard in pneumonia [26,27].

Heart murmurs are typically heard in patients with VHD, diseases that are more prevalent in an ageing population. While most cases of significant aortic stenosis are associated with a detectable murmur, other heart valve diseases may be hard to detect with a stethoscope [28]. Murmurs are also often heard when no VHD can be found,—so-called innocent murmurs [28]. Hearing a heart murmur in primary care often leads to referral to a cardiologist for echocardiography, a resource-intensive examination, frequently concluding that the murmur is innocent and not associated with a significant VHD. Innocent murmurs are particularly frequent in preschool children [29]. Referrals of children to cardiologists are increasing, and in one center the finding of innocent murmurs rose from 60% to 84% between 2007 and 2013 [30]. Low reliability of murmurs as a clinical sign may also be a problem, but compared to abnormal lung sounds, heart murmurs are more stable over time, although influenced by the workload for the heart during activity. There is variation between observers rating the same cardiac sounds, but less than has been found for lung sounds, and the variation seems to be less prominent among experienced listeners [31].

## Could a stethoscope become a better diagnostic tool when turning ‘smart’?

Machine learning technology enables computers to detect abnormal chest sounds based on expert annotations [1]. Physical properties of the sounds like frequency, amplitude, and timing within the respiratory or cardiac cycle can also be identified, details that may be hard to recognize with a traditional stethoscope. By ‘deep learning’, machines are also able to compare recorded chest sounds with various clinical endpoints such as disease entities and disease severity [32]. For such algorithms to be robust, they need to be trained on thousands of sound files from both healthy individuals and patients with chest diseases. Output from algorithms is based on probability scores. Although fallible, algorithms have been shown to make fewer errors than most clinicians. In the study by Prince and coworkers, the algorithm detected structural heart disease with a sensitivity of 88%, whereas the average detection rate among clinicians was 77%. The algorithm detected all cases with aortic stenosis, but overdiagnosis was common (with a specificity of 74%) [3]. Also, reports on algorithms for pathological murmurs in children have been promising [30,33]. Algorithms identifying crackle features have shown high accuracy for detecting interstitial lung disease (ILD) such as idiopathic pulmonary fibrosis [34]. A smart stethoscope can also help caregivers who monitor patients with an established diagnosis. Parents of children with asthma record lung sounds, transmit the recording to their physician, or use AI support to help manage their child’s condition [35,36].

Smart stethoscopes do not register the depth of the patient’s breathing or whether the heart beat is influenced by psychological stress. Clinicians will still depend on supplementary findings from history and biomarkers to enhance the diagnostic value of auscultation. Cardiac sounds and lung sounds are usually just small pieces of the diagnostic puzzle. Many of the practical challenges associated with the traditional stethoscope will not be resolved with a smart stethoscope. The stethoscope’s headpiece needs to be optimally positioned, and the breathing instructions to the patient need to be appropriate. Ambient noise will remain a source of interference; listening is often done in a busy environment, and scratching of the stethoscope on the patient’s skin may be distracting. Humans are adept at selective listening in a noisy environment, for instance focusing on a specific conversation when other talking is going on in the room (the cocktail party effect) [37]. This capability is valuable when ambient noise during auscultation needs to be filtered out. Can smart stethoscopes learn to be equally good?

The smart stethoscope will certainly become a stronger diagnostic tool than the traditional stethoscope. However, with the limited value of auscultation for diagnosing most chest diseases, one may question its utility when checking asymptomatic subjects. Listening for a heart murmur for detecting congenital heart defect in newborns [29] and among the elderly for a possible aortic stenosis are relevant screening procedures that could be done more efficiently with smart stethoscopes [28]. One large study from primary care found a somewhat higher incidence of valvular heart disease among GPs using a smart stethoscope compared to a reference group of GPs [38].

## Can smart stethoscopes lead to decreased auscultation skills?

While a decrease in performance of lung [39] and heart auscultation [40] predates the era of smart stethoscopes, there is now the risk of never even acquiring auscultation skills because of deference to this new and rapidly improving technology. Unless there is an educational component, i.e. a feedback loop by ‘machines as teachers’ [32], clinicians risk perceptual atrophy and will not build necessary expertise in pattern recognition [41]. The principle of ‘use it or lose it’ may prove applicable here [41]. The risk of AI-induced ‘deskilling’ may be further compounded should institutions require the incorporation of ‘objective’ data from smart stethoscopes in hospitals and clinics. Deskilling due to reliance on AI interpretations has been regarded as a challenge in several medical fields [41], and auscultation may prove particularly vulnerable given its dependence on perceptual skills. However, upskilling can also occur when a clinician with auscultatory competence uses AI-assisted interpretation as a reflective tool, comparing machine output against clinical perception to sharpen diagnostic acuity. This concept of a collaboration of humans and AI is drawing much attention, particularly with regard to keeping AI-algorithms trustworthy [42]. Keeping human experts ‘in the loop’ is also recognized by the developers of AI methods in the medical field [43]. *C*oncerns and promises we believe are linked to smart stethoscopes are shown in Table 1.

**Table 1.** Concerns and promises linked to smart stethoscopes.

Concerns	Promises
May lead to decline in auscultation skills	Helps clinicians with hearing difficulties
Dependence on digital networks	Reduced dependence on individual auscultation skills
Reduced clinical challenge and engagement for most clinicians	Recordings enable longitudinal comparison of chest sounds
Algorithm output may be disruptive and time-consuming	AI-generated interpretations provide immediate diagnostic support
Risk of clinical decisions being delegated to the algorithm	Valuable tool for medical education and clinical/epidemiological research

## Advantages of the old stethoscope

In the world market for stethoscopes the non-electronic versions still constitute the majority (Figure 2). The introduction of smart stethoscopes may entail increased costs, particularly when linked wirelessly to electronic health record systems. We need the traditional or a simple electronic stethoscope when internet connectivity is unavailable. In some clinical situations, the traditional stethoscope might be more functional. Auscultation is often done as a rapid check to ensure that the heart and lungs sound normal, in situations when the risk of serious disease is low and remains low even with a positive finding. How much time and attention should be devoted to chest auscultation in such situations? The comparison of one’s own normal findings with the smart stethoscope’s interpretations could be disturbing and a waste of time. In a noisy acute setting when the physician wants to urgently find out why the patient is severely short of breath, auscultation with a traditional stethoscope, after percussion of the lungs, will usually be sufficient for verifying a collapsed lung, a severe asthma attack, or pulmonary edema. Furthermore, clinicians cherish their traditional stethoscope and take professional pride in detecting signs of disease through auscultation. This interest in hearing and interpreting heart and lung sounds needs to be fostered through regular practice, if the clinician’s proficiency in chest auscultation is to be strengthened.

**Figure 2.**
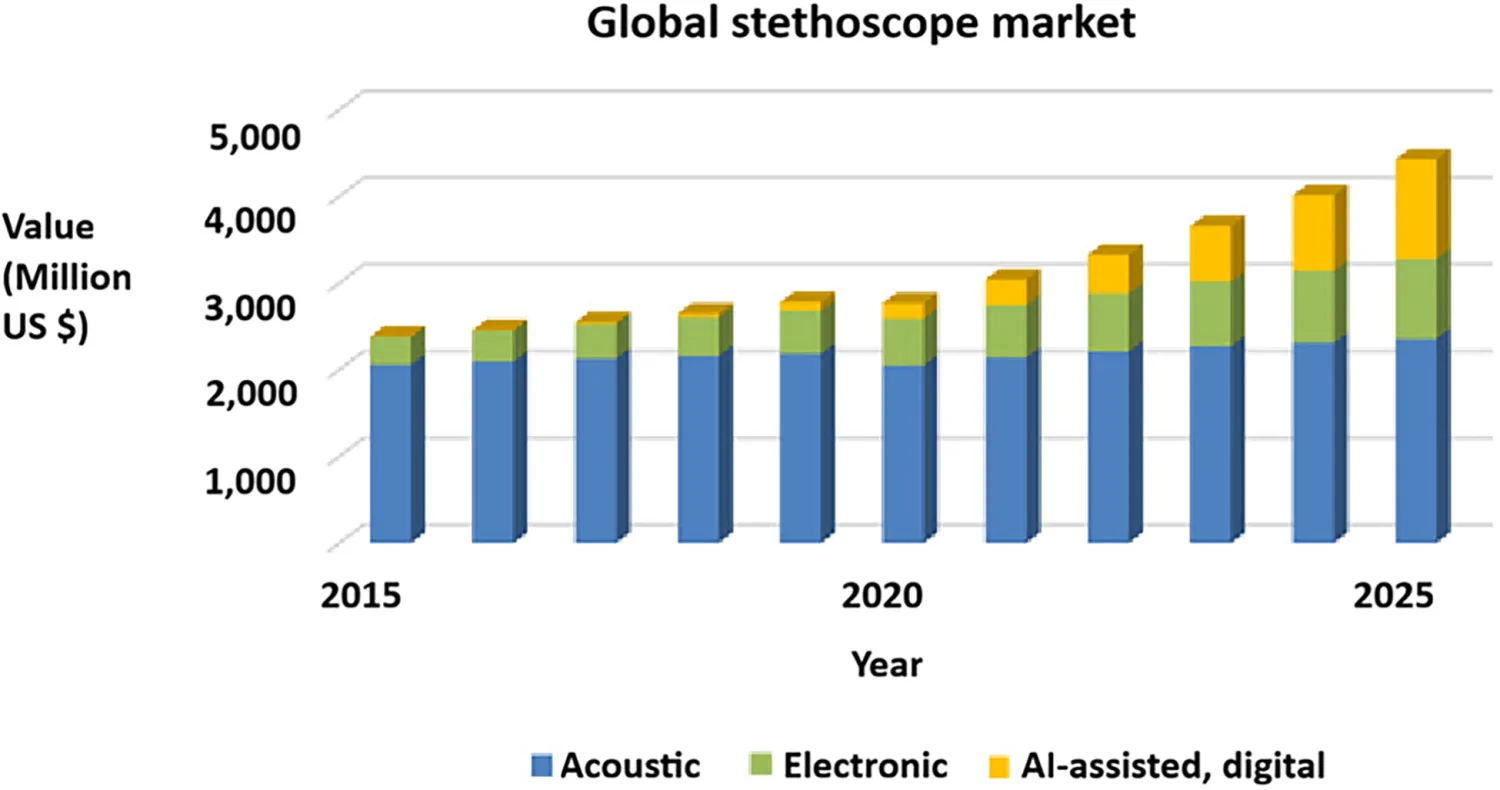
Global stethoscope market. *Sources*: data collected (with support from Claude/Anthropic) from Grand View Research ‘Stethoscope Market’ reports (2020, 2024); MarketsandMarkets ‘Digital Stethoscope Market’ (2023); Allied Market Research (2022); Mordor Intelligence (2024).

## Chest auscultation and the doctor-patient relationship

During auscultation, the examining physician tries to learn more about the patient’s situation, while the patient stays attentive to any comment from the physician on what has just been heard. Touch, even by a technical device when in the hands of the examining physician, may thus contribute to physician - patient communication [44]. Insofar as technological developments of the stethoscope help hearing and understanding in a straightforward way, technology is the physician’s best friend. Less attention needs to be given to concluding what has actually been heard, while more can be spent on the clinical implications of the findings and on interacting with the patient. When the stethoscope becomes truly intelligent, proposing further diagnostic routes, actual diagnoses and even treatments, it is no longer an extension of the physician’s personal competence, but virtually a third party in the consultation, thereby altering its dynamics. The clinical reasoning, where the symptom presentation, clinical findings, and the physician’s initial hypotheses are weighed against the suggestions provided by AI, takes place right in front of the patient. In principle, this resembles the situation when a physician consults clinical guidelines, but in reality, it might pose a real challenge to the rapport between physician and patient. Within seconds, a judgment has to be made for which the physician is fully responsible, yet the smart stethoscope may give suggestions that are beyond the comprehension of the physician. Such an imbalance may incline the physician to resign from responsibility, becoming merely an administrator of medical information technology [41]. The relationship may become drained on its hands-on contents, leading to an auscultation practice that is less challenging, less inspiring, and ultimately carried out with less engagement and clinical confidence.

## Smart stethoscopes for teaching and research

The smart stethoscope may bring teaching of auscultation to a higher level [32]. At the bedside, high quality recordings of chest sounds can be displayed and discussed in small groups of students. Also in lectures, demonstrations of relevant sound files may prepare students for their future clinical examinations. AI linked to the stethoscope may also provide epidemiological knowledge on probabilities of relevant diseases, knowledge that will inform future interpretation of sounds heard with a traditional stethoscope.

The smart stethoscope will be useful in medical research. Until recently, researchers have classified every sound file” manually”, based on careful listening and study of spectrograms. More than one rater has been needed to reduce the subjectivity of the annotation. This dependency on expert-labelled data has been both time-consuming and costly. We still need more knowledge of cardiac sounds and lung sounds, to gain a detailed understanding of the clinical significance of various sound characteristics. This knowledge will benefit not only algorithm development but also clinicians using traditional stethoscopes.

## Conclusion

Smart stethoscopes will prove to be more accurate and reliable instruments for diagnosing heart and lung diseases than the traditional stethoscope, but will also be accompanied by unwanted side effects. We might experience deskilling of auscultation proficiency and clinical reasoning when we rely on interpretations from AI. The old one, low-priced, easy to use and independent of digital networks, has its advantages and will remain the most commonly used stethoscope in the foreseeable future. Many users around the world will need to keep up or improve their proficiency in chest auscultation, and proficiency will also be needed when clinicians reflect upon AI outputs. Fortunately, smart stethoscopes will themselves serve as valuable tools for developing that proficiency.
